# Vascularized versus nonvascularized free fibular grafts in reconstruction of post-traumatic critical long bone defects, a comparative study

**DOI:** 10.1186/s10195-026-00923-9

**Published:** 2026-05-24

**Authors:** Amro A. Fouaad, Abdel-Hakiem Massoud, Mohamed Shaban, Mahmoud Abdel-Naby, Mohamed I. Abulsoud

**Affiliations:** Present Address: Al-Azhar University, Cairo, Egypt

**Keywords:** Fibula, Graft, Vascularized fibular graft, Nonvascularized graft, Critical bone defect, Long bone reconstruction, Orthopedic surgery

## Abstract

**Background:**

Reconstruction of critical-sized long bone defects is a complex orthopedic challenge. Free fibular grafts, vascularized (FVFG) and nonvascularized (NVFG), are established reconstructive options, but comparative clinical outcomes remain uncertain.

**Purpose:**

To compare clinical and radiological outcomes of FVFG and NVFG in patients with post-traumatic critical bone defects more than 10 cm.

**Methods:**

A randomized controlled trial was conducted with 50 patients assigned equally to FVFG or NVFG groups. The primary outcome was time to union, while secondary outcomes included graft hypertrophy, functional scores (DASH and LEFS), complication rates, and donor site morbidity.

**Results:**

The mean time to union was 5.78 months in the FVFG group and 6.17 months in the NVFG group, showing no statistically significant difference (*p* = 0.447). Rates of graft hypertrophy, functional recovery, and complications were comparable between the groups.

**Conclusions:**

Both FVFG and NVFG provide effective reconstruction for critical bone defects, with nearly similar healing times and functional outcomes. NVFG may represent a less complex alternative in selected cases.

Level of evidence I.

## Introduction

Critical-sized bone defects, particularly those greater than 6 cm in long bones, present a formidable challenge in orthopedic surgery. Such defects often result from high-energy trauma, infection, tumor resection, or delayed fracture healing, demanding complex reconstructive interventions to restore bone continuity and function. Various techniques have been developed to address these defects, including bone transport, induced membrane techniques, and bone grafting [1, 2].

Among bone grafting options, fibular grafts have gained wide acceptance owing to their favorable structural and biological properties. The fibula provides a cortical strut suitable for load-bearing reconstruction and, when harvested as a vascularized graft, maintains its blood supply to enhance osteogenic potential and incorporation. Free vascularized fibular grafts (FVFG) have become a preferred method in extensive and complex defects owing to their biological advantages of live bone transfer. However, FVFG is technically demanding and requires microsurgical expertise [3, 4].

Conversely, nonvascularized fibular grafts (NVFG) are easier to harvest and apply, relying on revascularization through creeping substitution from the host bed. While less complex surgically, they have been traditionally considered less reliable for large defects or compromised biological environments [5].

Despite the theoretical benefits of FVFG, clinical studies have yielded variable results when comparing vascularized and nonvascularized fibular grafts, especially in critical bone defects exceeding 10 cm. With limited evidence from randomized controlled trials, the optimal graft choice remains unclear.

This study aims to compare the clinical and functional outcomes of free vascularized versus nonvascularized fibular grafts in the management of post-traumatic critical bone defects of long bones measuring 10 cm or more. Primary end points include time to union, while secondary outcomes assess graft hypertrophy functional recovery and complication rates. The findings intend to inform surgical decision-making by clarifying the relative merits of these grafting techniques in complex reconstructive scenarios.

## Materials and methods

The current study is a prospective randomized controlled study (computer generated random sequence) including 50 patients aged 18–65 years, all presenting with post-traumatic critical bone defects measuring 10 cm or more in long bones, including the humerus, femur, and tibia, with age group between mean (31.20 ± 9.48) in the vascularized group, and (36.92 ± 9.41) in the nonvascularized fibula group. Both sexes were shared in the study, 36 males and 14 females were included. Post-traumatic defects occurred in 27 patients, and post traumatic infection occurred in 23 patients. Both the upper limps and lower limbs were included in the current study; 42 upper limb defects and 8 lower limb defects were included. Patients with systemic vascular diseases, post-tumor reaction, or soft tissue defect contraindications to surgery were excluded. Institutional ethical approval was obtained prior to study commencement, and informed consent was obtained from all participants. Patients were randomized into two equal groups of 25 patients each using a sealed envelope technique. Group A received free nonvascularized fibular grafts (NVFG), while group B received free vascularized fibular grafts (FVFG).

### Preoperative evaluation

All patients underwent thorough clinical examination and routine laboratory investigations. Radiographic assessment included standard anteroposterior and lateral views of the affected limb to measure defect size. For FVFG candidates, vascular assessment was performed using Doppler ultrasonography and computed tomography (CT) angiography to evaluate donor and recipient vessel patency.

*Surgical Technique*: all patients received intravenous antibiotics perioperatively at 1 h.

**Fig. 1:**
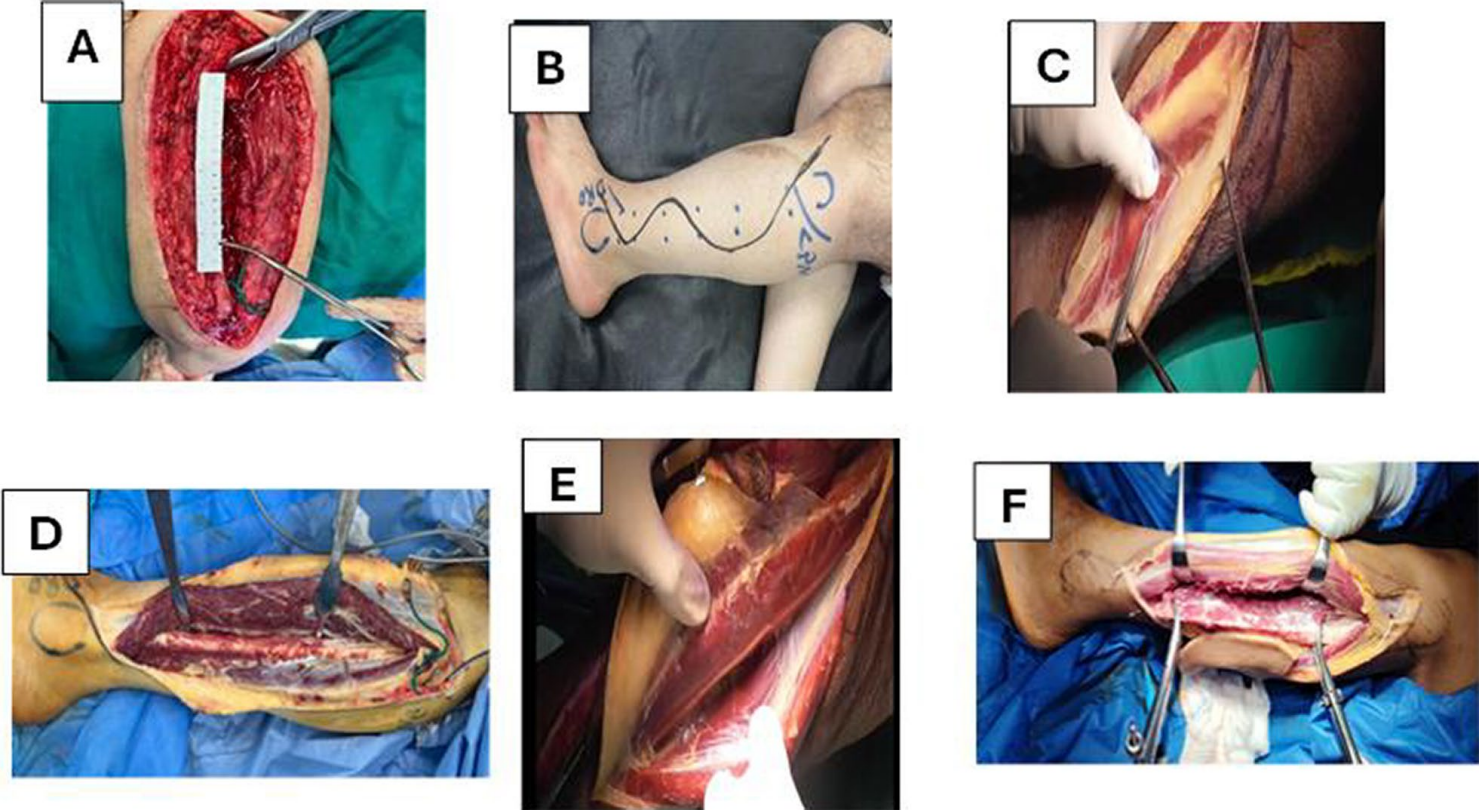
**A** Recipient site preparation (defect size-recipient vessels exploration artery and vein), **B** skin marking for free vascularized fibula harvesting, **C** start anteriorly by elevating peroneal muscles and identify skin perforator, **D** complete dissection until elevate anterior muscle group to reach interosseous membrane, **E** and **F** separate soleus muscle form flexor hallucis longus muscle

**Fig. 2:**
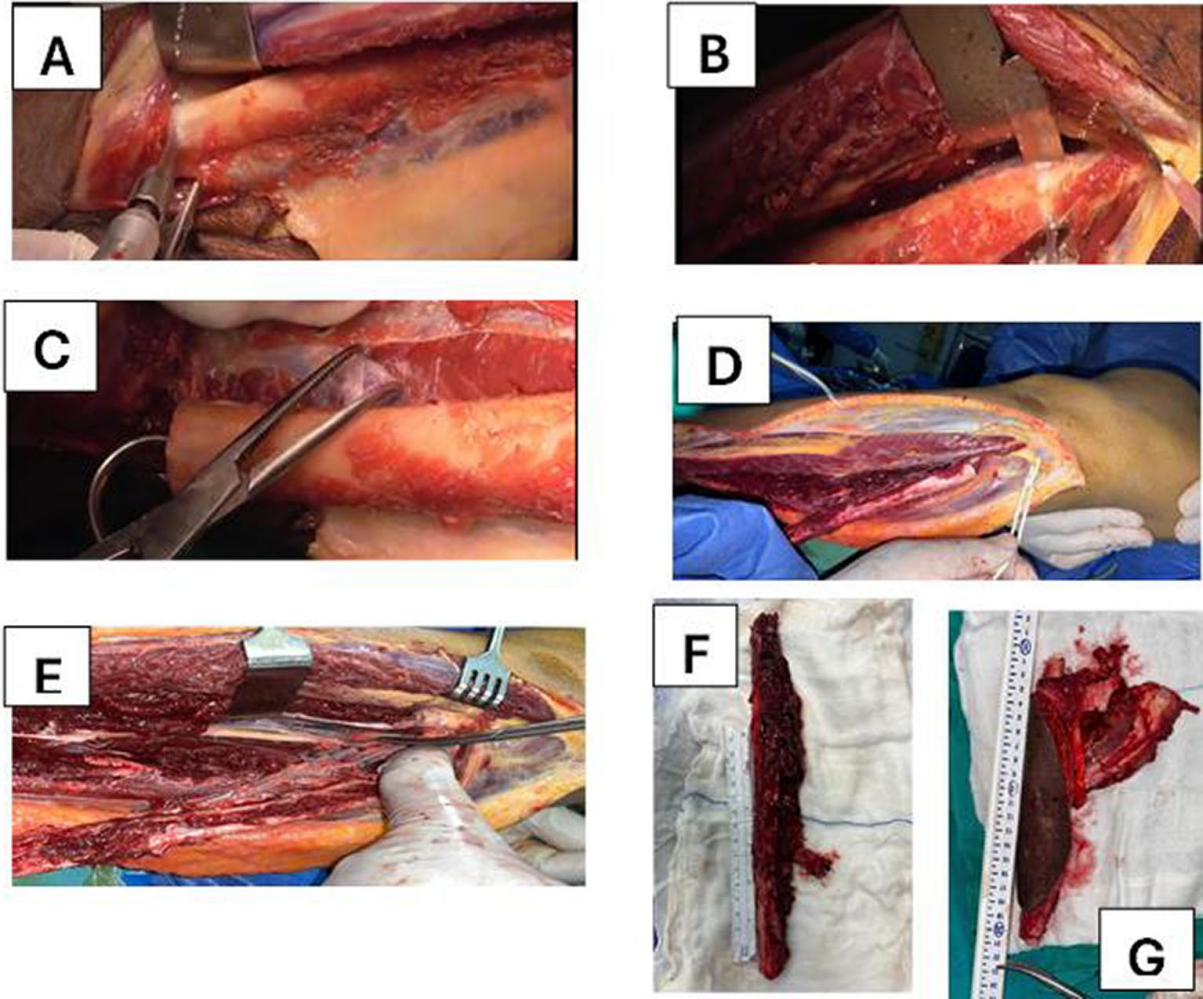
**A** Osteotomy of fibula done distally, **B** proximal fibular osteotomy, **C** distal run off identification, ligated and cotted, **D** complete separation of tibialis posterior muscle, flexor hallucis longus with preservation of pedicle, **E** complete dissection until reach tibia peroneal trunk (occlusion by vascular clamp and deflate tourniquet and check vascularity of limp before ligation and cutting), **F** free fibula with periosteal coverage and vascular pedicle, **G** free fibula with skin paddle and muscle

**Fig. 3:**
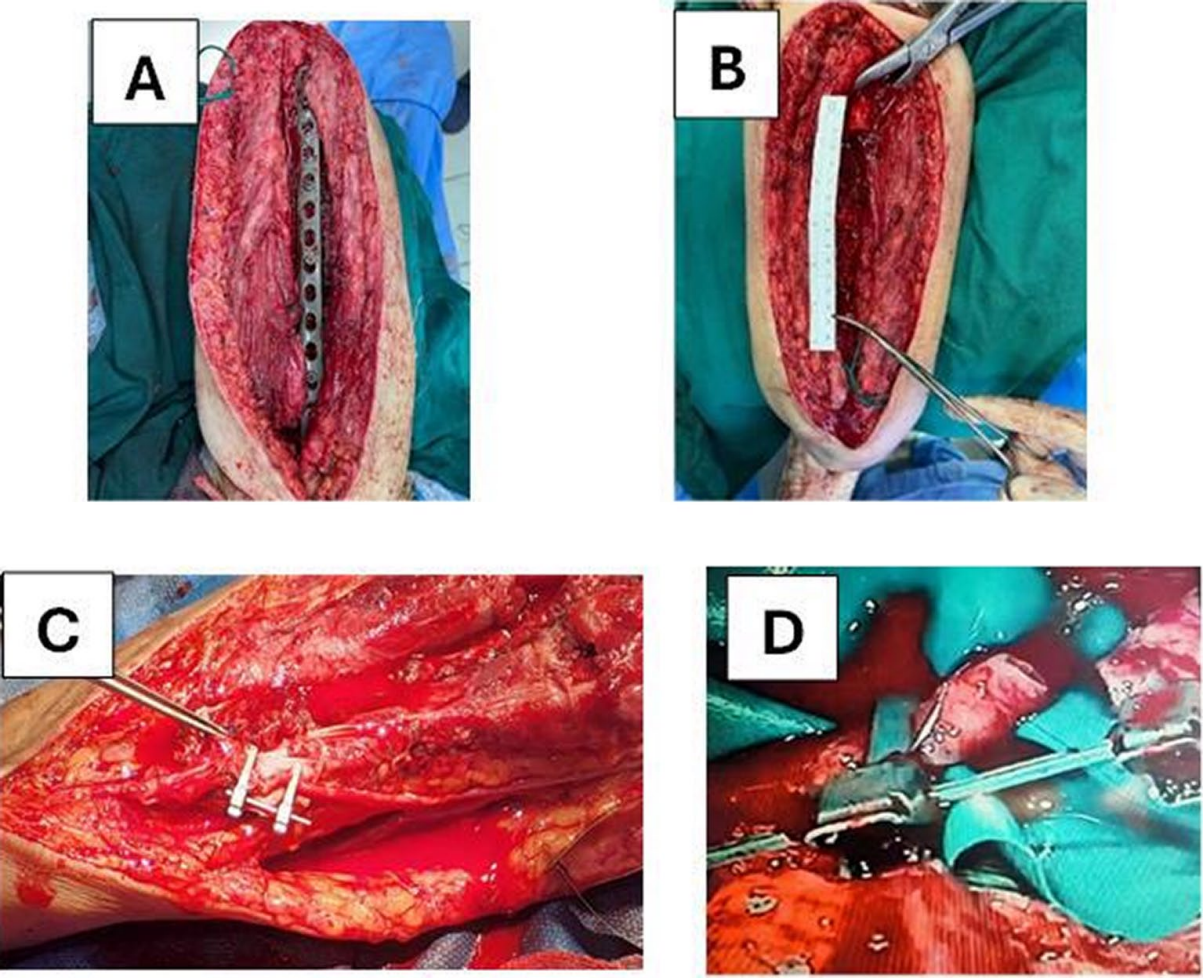
**A**, **B** Defect size measurement and preparation of graft and fixation by locked plate and screws, **C**, **D** anastomosis of vessels under microscope

*FVFG group*: after exploration and preparation of the donor side, debridement and measurement of defect size was performed(Fig. 1A). Under tourniquet and draping, the vascularized fibular graft was harvested from the ipsilateral or contralateral leg through an anterolateral approach (Figs. 1 B–D, F, 2 A–D, F, G). The graft was fixed with plates and screws; microvascular anastomosis was performed between peroneal vessels and donor vessels under the operating microscope (Fig. 3 A–D).

**Fig. 4:**
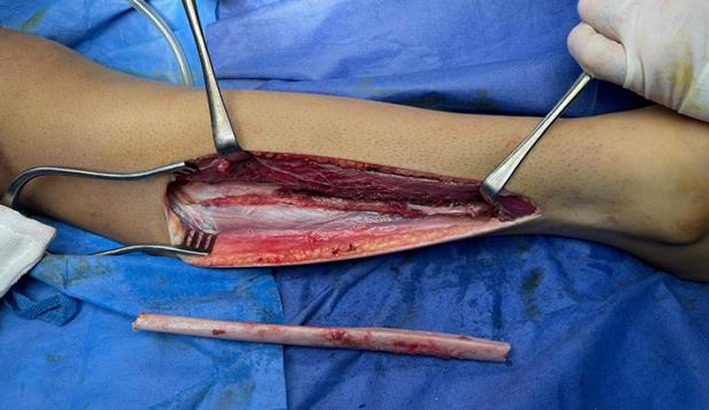
Lateral approach for harvesting free nonvascularized fibula subperiosteally with preservation of periosteum

*NVFG group*: a similar fibular segment was harvested without microvascular dissection. The graft was shaped and fixed to the defect site by standard orthopedic fixation techniques (Fig. 4).

### Postoperative care

Postoperative observation of vascularity of skin paddle for at least 7 days at ICU for group 1 vascularized fibula, and observation for early complications. The affected limb was immobilized using appropriate bracing or casting for 6 weeks, followed by gradual initiation of passive and active range of motion exercises. Weight-bearing and/or active exercise was delayed until radiological evidence of union was noted, with partial weight-bearing introduced progressively thereafter.

### Outcome measures

*Primary outcome*: time to radiological union defined as the appearance of bridging callus across the defect on at least three cortices on radiographs and no localized tenderness.

*Secondary outcomes*: graft hypertrophy assessed by hypertrophy index on serial x-rays, El Gammal Hypertrophy index formula, *H*_I_ = *D*_f_−*D*_i_ on *D*_i_ × 100. *D*_f_ diameter of fibula graft at follow up, *D*_i_, diameter of fibula immediately postoperative, interpretation from < 0% graft atrophy or resorption, to > 100% significant hypertrophy [3].

Functional outcomes measured by disabilities of the arm, shoulder, and hand (DASH) score for upper limb defects and lower extremity functional scale (LEFS) for lower limb defects, and perioperative complications including infection, graft fracture, and donor site morbidity.

## Results

A total of 50 patients completed the study with 25 patients in each group: free vascularized fibular graft (FVFG) and nonvascularized fibular graft (NVFG). Both groups were comparable in terms of baseline characteristics including age, sex distribution, defect size, and bone involved. Age, sex, and pathological cause were included (Table 1). Region and specific bone, defect length, and fixation method were also included. The upper limb cases were more prevalent than those for the lower limbs, especially the humerus, which is the most common affected bone in the current study. No significant difference between both groups with regards to age, sex, defect length, pathological cause, and fixation method was observed. Table 2 describe outcome measures, union time, hypertrophy, complications, donor side morbidity, DASH score and LEFS score.

**Table 1:** Demographic data of patients

Variable	Non-vascularized (*n* = 25)	Vascularized (*n* = 25)	Test	*P* value
Sex (male/female)	18 (72%)/7(28%)	18 (72%)/7(28%)	Chi^2^	1.000
Age (years)	36.9 ± 9.4	31.2 ± 9.5	*t*	0.037*
Pathology (trauma/infection)	17 (68%)/8(32%)	10 (40%)/15(60%)	Chi^2^	0.047*
Site (upper limb/lower limb)	17 (68%)/8(32%)	25 (100%)/0	Chi^2^	0.004*
Specific bone site	Humerus 15, Radius 2, Ulna 0, Femur 7, Fibula 1	Humerus 11, Radius 5, Ulna 8, Radius + Ulna 1, Femur 0, Fibula 0	Chi^2^	< 0.001*
Defect length (cm)	11.9 ± 1.6	12.5 ± 2.0	*t*	0.275
Fixation method (A/B)	25 (100%)/0	24 (96%)/1 (4%)	Chi^2^	1.000

* means statistically significant

**Table 2:** Outcome measures

Outcome	Nonvascularized	Vascularized	Test	*P* value
Union time (months)	6.2 ± 2.0	5.8 ± 2.0	Mann–Whitney *U*	0.447
Hypertrophy (yes/no)	6 (24%)/19 (76%)	10 (40%)/15 (60%)	Chi^2^	0.225
Complications (early/late)	3 (12%)/2 (8%)	10 (40%)/7 (28%)	Chi^2^	Early: 0.024*
Donor site morbidity	2 (8%) (toe extension loss)	4 (16%) (cramps/numbness)	Chi^2^	0.082
DASH score	38.1 ± 11.0 (*n* = 17)	43.6 ± 15.3 (*n* = 25)	Mann–Whitney	0.276
LEFS score (nonvasc only)	69.0 ± 5.4 (*n* = 8)	–	–	–

* means statistically significant

The primary outcome, time to radiological union, showed a mean duration of 5.8 ± 2.0 months in the FVFG group compared with 6.2 ± 2.0 months in the NVFG group. The difference was not statistically significant (*P* = 0.447). Radiological signs of bone healing appeared similarly in both groups, with bridging callus formation evident on serial x-rays by 4–6 months postoperatively. Graft hypertrophy was observed in VFG in 10 patients (40%) of the 25 patients, was mild in 5 patients < 25%, moderate in 4 patients 25–50%, and significant in one case (more > 100%).

In the NVFG group, graft hypertrophy was observed in 6 (24%) of the 25 patients (6/25); however, mild hypertrophy (< 25%) was observed in one patient, moderate hypertrophy (25–50%) in 3 patients, and significant hypertrophy in 2 cases (more > 100%); this difference did not reach statistical significance (*P* = 0.225), suggesting similar biological remodeling between the two groups and that graft remodeling was not dramatically enhanced by vascularization in these samples. Functional outcomes were assessed by DASH (38.1 ± 11.0 (*n* = 17) in nonvascularized and (43.6 ± 15.3 (*n* = 25)) vascularized groups, showing slightly higher values in the vascularized group compared with the nonvascularized group; however, differences were not statistically significant. LEFS scores at 12 months postsurgery showed no significant differences between groups, with most patients regaining satisfactory limb function for daily activities.

*Complication rates were comparable* Minor complications such as superficial infection occurred in two patients in the FVFG group and three in the NVFG group. Graft fractures occurred in one patient within each group. No cases of total graft failure were reported. Two cases of loss of big toe extension in NVFG, muscle cramps in three cases of donor legs, and one case of numbness of the first web in FVFG suggest an increased donor side risk with vascularized grafts, although this finding was not clinically overwhelming.

Overall, both FVFG and NVFG achieved high success rates in the treatment of post-traumatic critical bone defects, demonstrating similar efficacy in promoting bone union and functional recovery without significant differences in complication profiles.

### Case presentation

**Fig. 5:**
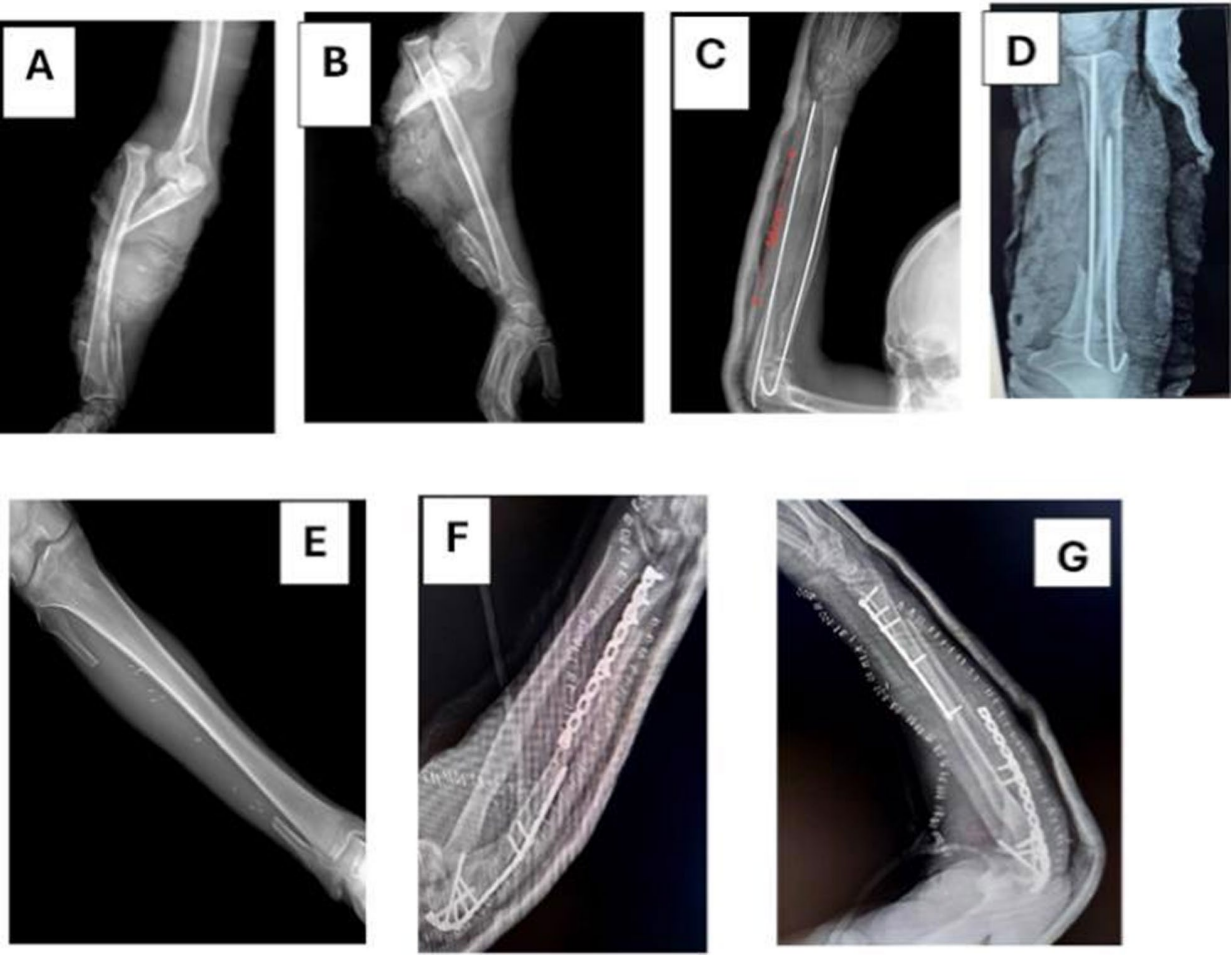
**A**, **B** Pre-x-ray, **C**, **D** 1st stage postpreliminary fixation by k-wire and reduction of radial head, **E** x-ray leg postharvest free fibula, **F**, **G** immediate postoperative x-ray

**Fig. 6:**
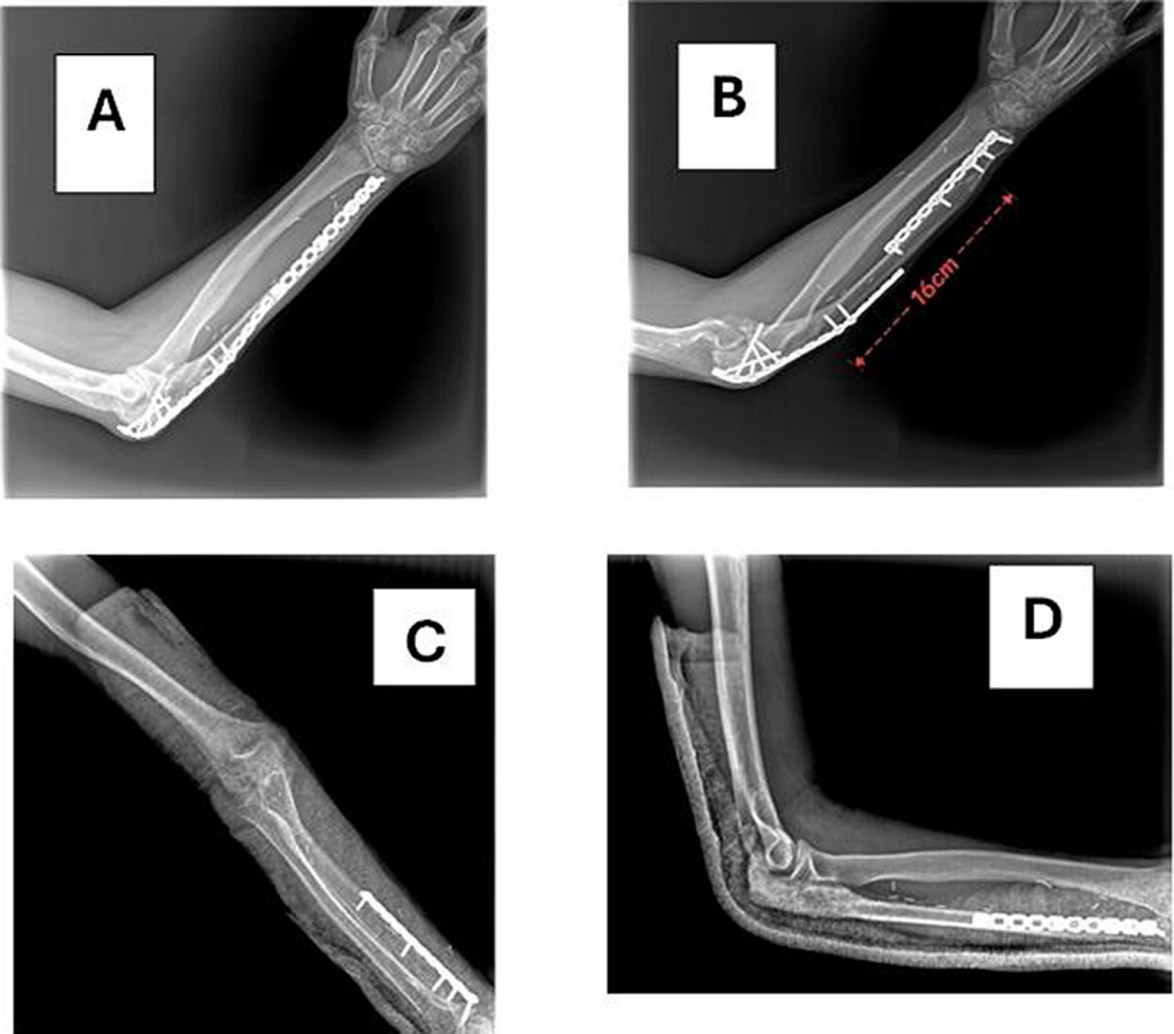
**A**, **B** Follow up after 1 year, **C**, **D** follow up after 1.5 years and removal of prominent proximal plate

**Fig. 7:**
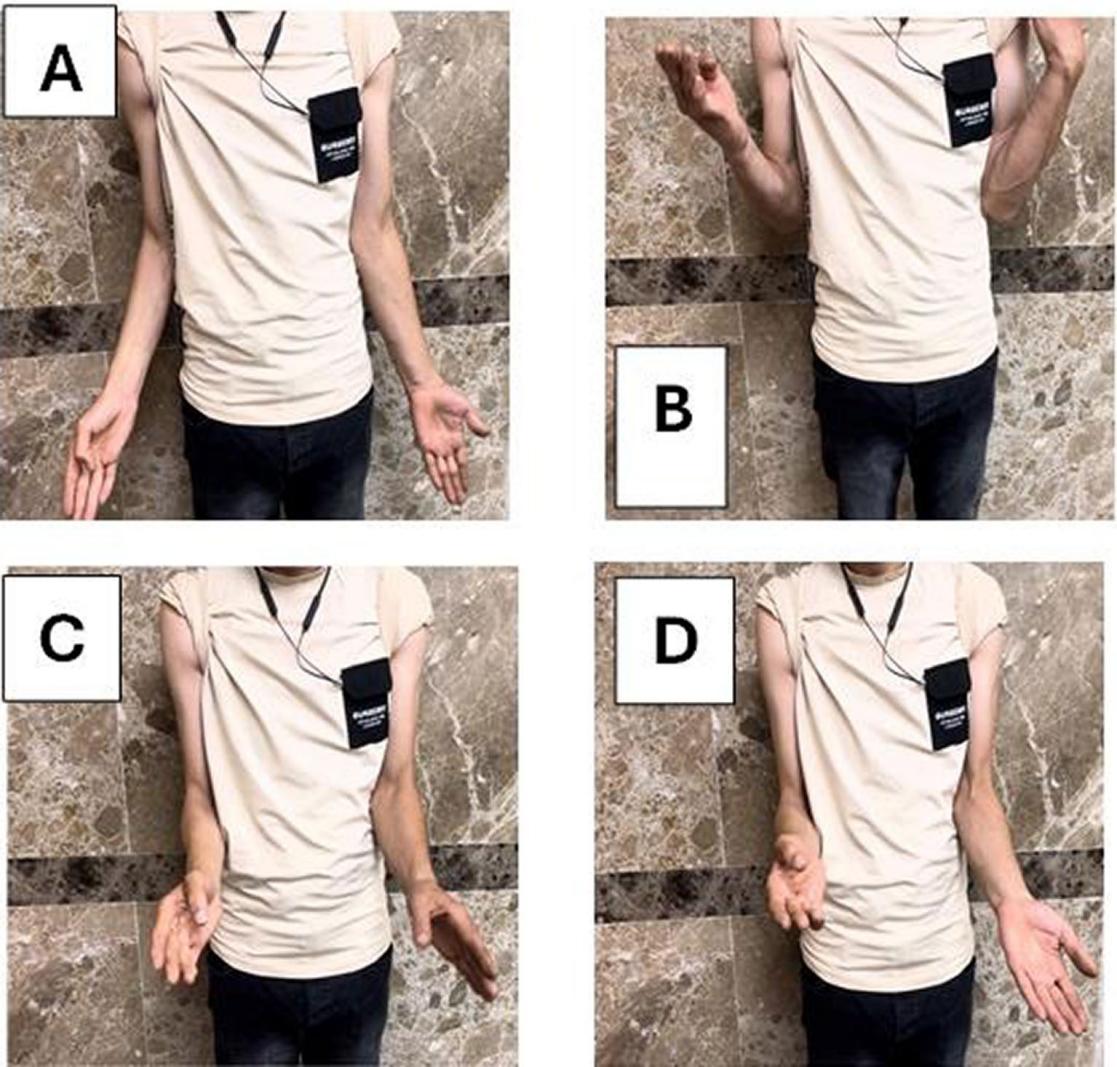
**A**, **B** Show range flexion and extension, **C**, **D** pronation and supination range of motion

*Case 1* Male patient, 29 years old, high energy trauma, 2nd degree open fracture with bone loss in the right ulna and radial head dislocation, debridement and preliminary fixation by k-wires, 2nd stage reconstruction by free vascularized fibular graft fixation by double plating, follow up 1.5 years (bone defect: 16 cm, time to healing: 4 months proximal and distal end (Figs. 5, 6, 7).

**Fig. 8:**
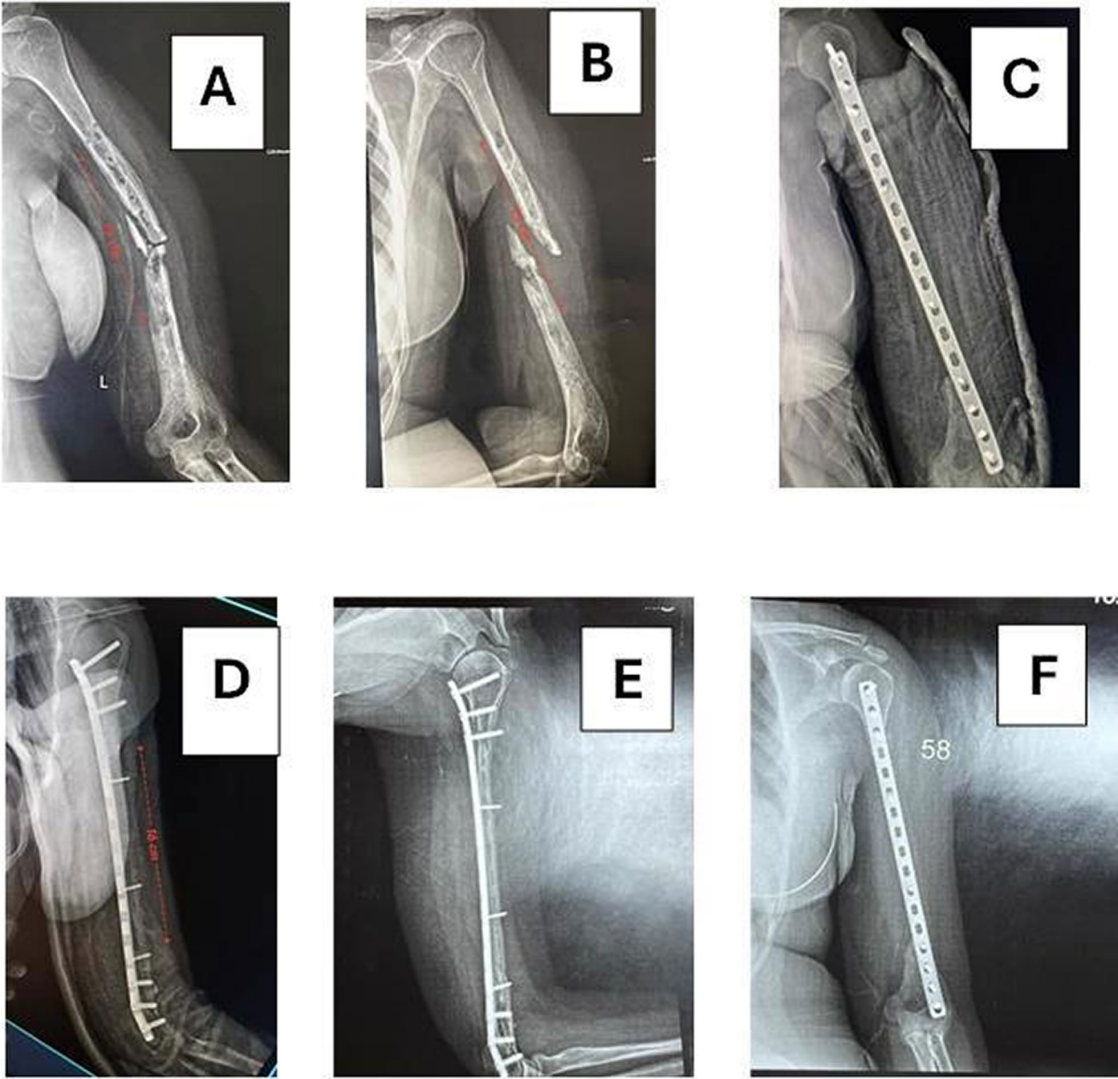
**A**, **B** Preoperative x ray before 1st stage, **C**, **D** immediate postoperative x ray, **E**, **F** last follow up x-ray after 1 year

**Fig. 9:**
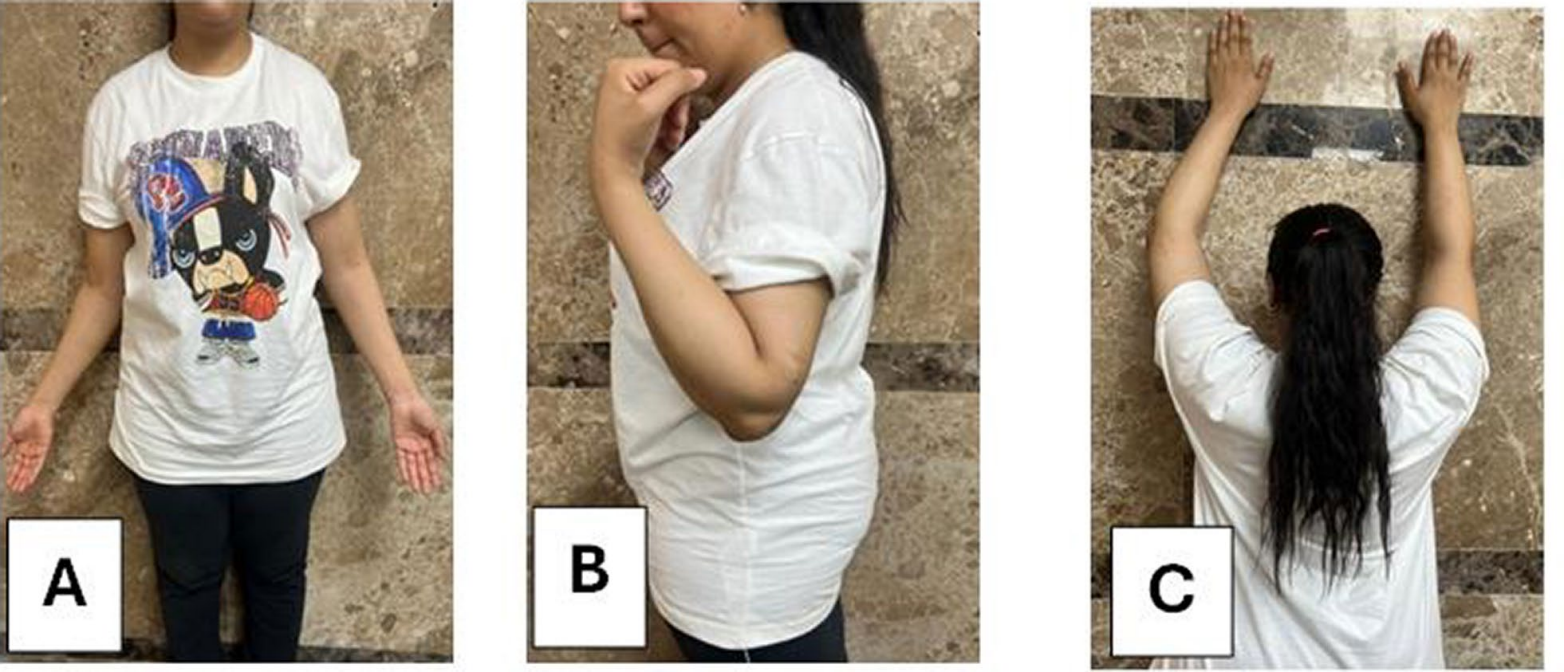
**A**, **B** Elbow range of motion extension–flexion, **C** shoulder abduction

*Case 2* A female patient, aged 23 years old, presented with an infected nonunion humerus following fixation of a simple shaft fracture with a plate and screws. Postoperatively, infected serial debridement occurred, leading to the removal of the plate and application of an external fixator. The infection did not improve after seven surgeries, leading to debridement and excision of the sequestrated bone and filling of the space using bone cement and Nancy nails as the first stage. After 2 months, the cement was removed and a free nonvascularized fibular graft was fixed by a long locked narrow DCP plate and screws. The defect size was 16 cm, and time to healing was 5 months (Figs. 8, 9).

**Fig. 10:**
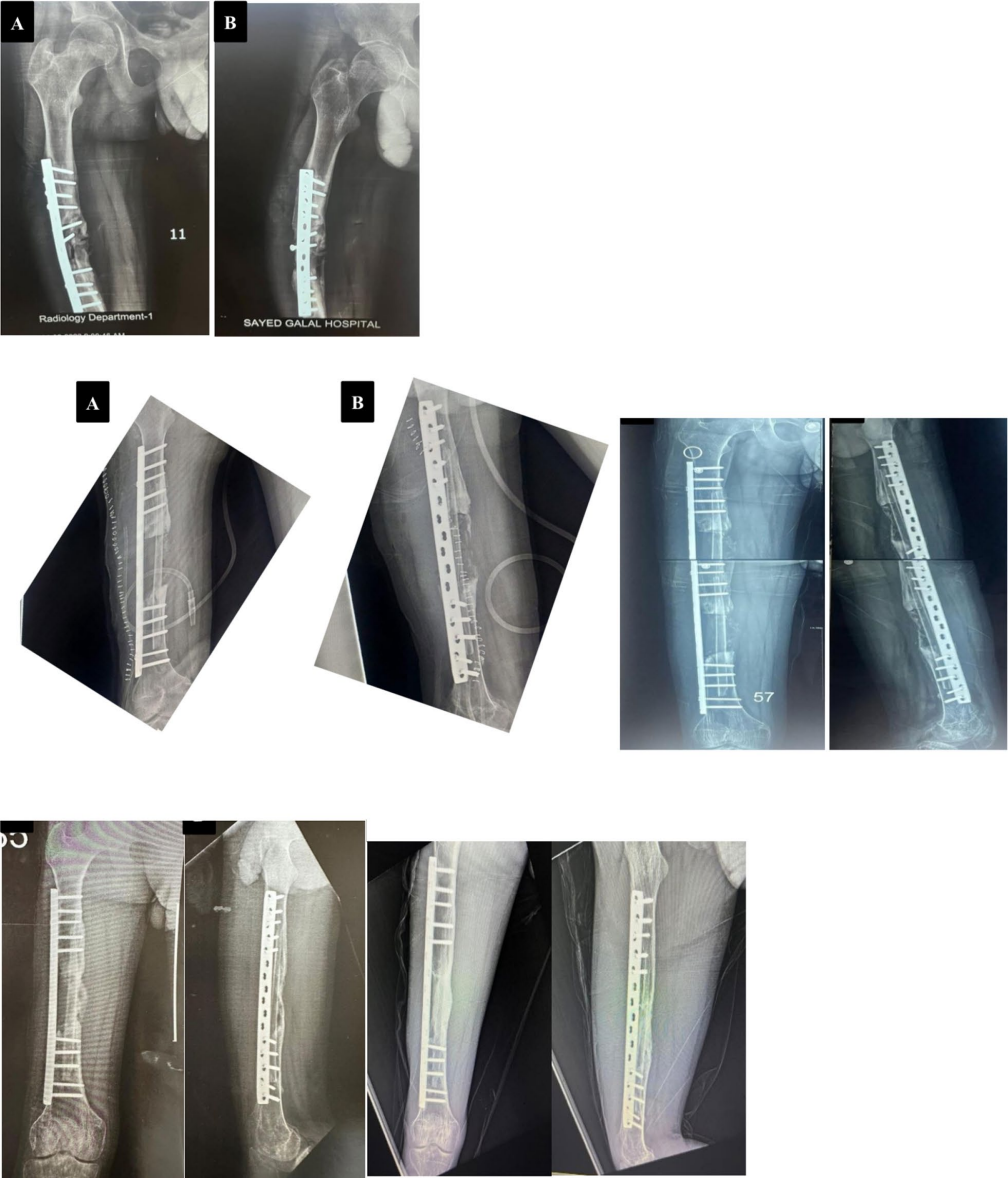
**A**, **B** Preoperative femoral mechanical failure and osteomyelitis, **C** immediately after operation, **D** after 6 weeks, **E** after 6 months, **F** after 12 months

*Case 3* A male patient, 18 years old, presented with post-traumatic femoral osteomyelitis. The first stage of treatment was debridement and the Masqul technique. At 2 months, second exposure and debridement was performed with a defect size of 10 cm. NVFG was performed with fixation by a locked broad plate. Compete consolidation with hypertrophy > 50% without complications for the recipient or donor was achieved at 9 months (Fig. 10).

## Discussion

The management of critical-sized bone defects remains one of the most challenging areas in orthopedic reconstructive surgery. This study compared free vascularized fibular grafts (FVFG) and nonvascularized fibular grafts (NVFG) in post-traumatic long bone defects > 10 cm and found no statistically significant differences in union time, graft hypertrophy, functional recovery, or complication rates. These results correspond with recent evidence that questions the traditional 6 cm rule, which advocates for vascularized grafts for defects longer than 6 cm.

Historically, the belief that longer grafts require vascularization stems from limited early case series, but systematic reviews, including that by Allsopp et al. [6], demonstrate a lack of solid evidence supporting this rule. Their review revealed that vascularized grafts did not decrease time to union nor improve union rates significantly when compared with nonvascularized grafts for long-bone defects. Additionally, vascularized grafts carried higher revision risks owing to complications such as wound breakdown and mechanical failure. These findings align with our observation of similar union times (5.78 versus 6.17 months) and complications in both groups.

For instance, El-Gammal et al. [7] reported earlier union in FVFG compared with NVFG, especially in defects exceeding 6 cm or in the presence of infection. Similarly, Pho et al. [8] and Chen et al. [9] emphasized the role of vascularized grafts in enhancing early revascularization and osteogenesis. However, our results are consistent with reports by Delloye et al. [10], Qadir et al. [11], who found no major difference in long-term union rates between FVFG and NVFG when proper fixation and soft-tissue conditions were ensured without active infection. Recent studies have continued to support the utility of vascularized fibular grafts (FVFG), particularly in large, avascular, or previously infected defects. For example, Han et al. [12], reported superior union times with FVFG in defects > 6 cm, but also noted higher complication rates. Wang et al. [13] found that while FVFG achieved faster union, nonvascularized fibular grafts (NVFG) provided comparable long-term functional results in smaller defects (< 6 cm).

Ibrahim et al. [14] showed that with stable fixation and good recipient vascularity, NVFG can have union rates close to FVFG, aligning with our findings. Zhao et al. [15], in a meta-analysis, concluded that the clinical advantage of FVFG may be limited to selected cases, and NVFG remains appropriate in many scenarios. Our current study supports these more recent findings that question the universal superiority of FVFG and instead promote a case-based, individualized approach.

Graft hypertrophy is often used as a surrogate for biological activity and integration under mechanical loading. In the current study, hypertrophy occurred in 24% of NVFG cases and 40% of FVFG, but this difference was not statistically significant (*P* = 0.225). FVFG has traditionally been credited with greater remodeling capacity, suggesting that mechanical loading, soft tissue environment, meticulous dissection and fixation stability may be more critical determinants of hypertrophy than graft vascularity alone—a view supported by studies such as those by Minami et al. [16] and Doi et al. [17].

In both groups, hypertrophy occurred without statistically significant differences, while FVFG is expected to improve hypertrophy due to intrinsic vascularity, a theory also discussed in Liu et al. [18], Interestingly, early complications were more frequent in the FVFG group (40%) compared with NVFG (12%), and this difference was statistically significant (*p* = 0.024). This reflects the greater surgical complexity and technical demands of free vascularized grafts.

Although FVFG may be beneficial in complex or avascular environments, it carries higher operative risk, requires microsurgical expertise, and is associated with donor-site morbidity and longer surgical time, as described by Weiland et al. [19], Yajima et al. [20] and Mehdi et al. [21]. Consistent with the literature, we found higher early complication rates in the FVFG group (40%) compared with NVFG (12%) (*P* = 0.024).

The Masquelet-induced membrane technique has gained popularity for managing large bone defects. Compared with both FVFG and NVFG, it is less technically demanding, involves staged procedures, and can be used with autograft, allograft, or bone substitutes. Recent studies include: Pelissier et al. [22] reported union rates of 80–90% using the Masquelet technique in defects > 5 cm.

Giannoudis et al. [23] found no significant difference in union time between Masquelet and FVFG but highlighted increased infection control and cost-effectiveness with the Masquelet technique. Mauffrey et al. [24] emphasized the role of the Masquelet technique in infected defects, often with fewer donor-site complications. El-Alfy et al. [25] and Houdek et al. [26] showed that nonvascularized fibular grafts can successfully bridge defects exceeding 6–8 cm with satisfactory outcomes, provided optimal mechanical stability and biological environment are ensured.

Mukherjee et al. [27] conducted a similar comparative study and found no significant difference in union time or complication rates between vascularized and nonvascularized grafts in long bone defects, although the sample size was smaller.

Yin et al. [28] and Pho et al. [29] historically emphasized that vascularized fibular grafts have superior outcomes in defects > 6 cm, particularly in the presence of infection, poor vascularity, or soft-tissue compromise. However, in comparison with FVFG, the Masquelet technique requires two operations, and the second stage is time dependent. Also, no intrinsic vascularity wass present in the graft, potentially limiting remodeling in very large defects. Free vascularized fibular graft (FVFG) enhanced biological activity due to intrinsic blood supply, leading to Faster graft incorporation and remodeling, higher union rates in compromised biological beds (e.g., postinfectious, irradiated, or poorly vascularized areas), and greater resistance to infection, making it more suitable for septic or previously infected bone defects and allowing simultaneous transfer of soft tissue (skin paddle) when needed. However, its disadvantages are that it is technically demanding, requires microsurgical expertise and longer operative time, longer hospitalization, and potentially increased cost. Furthermore, it may not be feasible in all centers, especially in low-resource settings. Nonvascularized fibular graft (NVFG) is technically simpler and does not require microsurgery, has a shorter operative time and lower surgical cost, is suitable for centers without microsurgical capability, and is associated with less donor site morbidity and quicker recovery in some cases. However, this technique is not recommended in cases of a poor vascularity bed or active infection owing to slower remodeling and incorporation compared with FVFG in complex cases, which will be noted in the study limitations.

Although we ensured matched defect lengths and fixation methods, the nonrandomized nature and relatively small sample size may have limited our ability to detect subtle differences. Additionally, we did not stratify outcomes by bone type and comparison between upper limb nonweight-bearing and lower limb weight-bearing, or prior infection in the subgroup analysis, which could affect graft performance.

This study adds value by directly comparing FVFG and NVFG using matched groups and consistent surgical protocols. However, limitations include; small sample size, absence of long-term follow-up on graft durability. Several potential explanations account for this divergence. Many previous studies that demonstrated superior outcomes with FVFG included patients with very large defects (> 6–8 cm), poor vascularized beds (e.g., after infection, irradiation), severe soft tissue damage.

In our study, the mean defect size was relatively moderate (~12 cm), and most defects were not infected, with good local vascularity. This may have allowed NVFG to perform equally well owing to sufficient biological support from surrounding tissues. Supporting point: studies such as Wang et al. [13] and Ibrahim et al. [14] also found comparable outcomes in moderately sized defects when soft tissue quality was preserved.

Our study found that both techniques achieved similar healing times, challenging the conventional preference for FVFG. This stands in contrast to most published studies, particularly older and traditional literature, which generally report faster union, higher success rates, and enhanced remodeling in FVFG, such as the study conducted by Houdek et al. [26].

Both groups in our study received rigid fixation with appropriate implants, minimizing micromotion and allowing biological incorporation. Adequate mechanical stability is a critical determinant of bone healing, especially in NVFG, where vascular supply relies on creeping substitution. This is consistent with findings by Liu et al. [18] and Minami et al. [16], who emphasized biomechanical environment over vascularity in graft incorporation and hypertrophy.

Our patients were treated under a standardized postoperative rehabilitation protocol, including controlled weight bearing and physiotherapy. Such consistent care may have minimized differences between groups and promoted graft remodeling and union in both. Unlike older studies where NVFG failure was often linked to poor technique or handling, our surgical team applied strict techniques for harvesting, shaping, and press-fitting the graft, reducing necrosis and increasing incorporation rates. Much of the earlier literature focused on FVFG owing to its novelty and technical appeal. There may be publication bias favoring positive results with vascularized grafts, particularly from high-volume microsurgical centers. Moreover, some older studies did not control for confounding factors such as defect size, infection, or fixation methods, which could overstate the advantage of FVFG.

Many comparative studies in the past were small or retrospective, increasing variability and bias. By using matched cohorts and consistent surgical protocols, our study attempted to reduce heterogeneity, possibly leading to more balanced and realistic results in essence. The lack of significant difference in our study is likely due to: appropriate case selection, post-traumatic or post-osteomyelitis, optimal fixation, high-quality surgical technique, moderate defect sizes, good recipient vascularity, and possibly a shift in clinical protocols that optimize NVFG outcomes in recent years.

Our findings do not refute the utility of FVFG, especially in complex or large defects, but rather emphasize that NVFG can achieve equivalent results in many standard trauma cases.

## Conclusions

The current study demonstrates that both free vascularized and nonvascularized fibular grafts are effective for reconstructing post-traumatic and post traumatic osteomyelitis, critical bone defects of > 10 cm in long bones. No significant differences were observed in time to union, graft hypertrophy, functional recovery, or complication rates between both groups. The comparable outcomes suggest that nonvascularized fibular grafting may serve as a less complex and resource-intensive alternative to vascularized grafting in selected patients. Surgical decision-making should consider patient-specific factors, surgeon expertise, and available infrastructure. Future larger-scale studies with longer follow-up are warranted to validate these findings and refine graft selection criteria.

## Data Availability

All data is available in the excel sheet personal, clinical and results, and is in compliance with the Declaration of Helsiniki. Start working at Work license start 4-2023, first patient at 5-2023 up to last patient at 5-2024, with license NO, Pat_3 med. Research ortho.00003, http://www.medicneazhar.edu.eg.
